# Impact of Spent Mushroom Substrate Combined with Hydroponic Leafy Vegetable Roots on *Pleurotus citrinopileatus* Productivity and Fruit Bodies Biological Properties

**DOI:** 10.3390/microorganisms12091807

**Published:** 2024-09-01

**Authors:** Ilias Diamantis, Marianna Dedousi, Eirini-Maria Melanouri, Eleni Dalaka, Paraskevi Antonopoulou, Alexandra Adelfopoulou, Seraphim Papanikolaou, Ioannis Politis, Georgios Theodorou, Panagiota Diamantopoulou

**Affiliations:** 1 Laboratory of Edible Fungi, Institute of Technology of Agricultural Products (ITAP), Hellenic Agricultural Organization—Dimitra, 1, Sofokli Venizelou, 14123 Lykovryssi, Greece; idiamantis@aua.gr (I.D.); mdedousi@aua.gr (M.D.); eimelanouri@aua.gr (E.-M.M.); parantonopoulou@gmail.com (P.A.); 2 Laboratory of Food Microbiology and Biotechnology, Department of Food Science and Human Nutrition, Agricultural University of Athens, 75 Iera Odos, 11855 Athens, Greece; spapanik@aua.gr; 3 Laboratory of Animal Breeding & Husbandry, Department of Animal Science, Agricultural University of Athens, 75 Iera Odos, 11855 Athens, Greece; elenidalaka@aua.gr (E.D.); alexandraadelfopoulou@yahoo.gr (A.A.); i.politis@aua.gr (I.P.); gtheod@aua.gr (G.T.)

**Keywords:** agricultural waste, mushrooms, antioxidants, lipids, protein, carbohydrates

## Abstract

Agricultural activities produce large quantities of organic byproducts and waste rich in lignocellulosic materials, which are not sufficiently utilized. In this study, alternative agricultural waste products, namely, spent mushroom substrate (SMS) from the cultivation of edible *Pleurotus ostreatus* mushrooms and the roots of leafy vegetables from hydroponic cultivation (HRL), were evaluated for their potential to be used as substrates for the cultivation of *Pleurotus citrinopileatus* and their effects on the quality, the nutritional value, the chemical properties (lipid, protein, carbohydrate, ash, fatty acid and carbohydrate composition) and the bioactive content (total phenolic compounds and antioxidant activity) of produced mushrooms. SMS and HRL (in different ratios with and without additives) and wheat straw with additives (WS—control) were used. During incubation, the linear growth rate of the mycelium (Kr, mm/day) was measured and used for screening. Mushroom cultivation took place in bags, where several characteristics were examined: earliness (duration between the day of substrate inoculation and the day of first harvest) and biological efficiency (B.E. %, the ratio of the weight of fresh mushrooms produced per dry weight of the substrate × 100). Furthermore, this study aimed to investigate the effect of the protein extract (PE) and carbohydrate extract (CE) of *P. citrinopileatus* after in vitro digestion (fraction less than 3kDa: PE-DP-3; digestate fraction: CE-D, respectively) on the expression of antioxidant-related genes in the THP-1 cell line. The results showed that mushrooms grown on SMS 50%-HRL 40% had the fastest growth (6.1 mm/d) and the highest protein and lipid contents (34.7% d.w.; 5.1% d.w.). The highest B.E. (73.5%), total carbohydrate (65.7%) and total phenolic compound (60.2 mg GAE/g d.w.) values were recorded on the control substrate. Antioxidant activity was observed in all extracts; the total flavonoid content was low in the samples, and the maximum total triterpene value was detected in SMS 80%-HRL 20% (9.8 mg UA/g d.w.). In all mushrooms, linoleic acid (C18:2) was the main fatty acid (above 60%), and fructose was the dominant individual saccharide. In the investigation of the regulation pathway, NFE2L2 gene expression was upregulated only in the SMS 60%-HRL 40% intervention during incubation with CE-D samples. Additionally, the transcription levels of antioxidant-related genes, SOD1, CAT, HMOX1 and GSR, were increased in the SMS 60–30% intervention. Compared to WS, the alternative substrates are observed to trigger a pathway concerning CE that may resist oxidative stress. This study supports the utilization of agricultural byproducts through sustainable and environmentally friendly practices while simultaneously producing high-value-added products such as mushrooms. Therefore, alternative substrates, particularly those containing HRL, could serve as natural sources of antioxidant potential.

## 1. Introduction

The rapidly growing global population, along with the expansion of the agricultural sector and food industries, has led to the annual generation of substantial amounts of agro-industrial waste. This waste is defined as the byproducts generated during the industrial processing of agricultural or animal products, as well as the waste resulting from agricultural activities [1,2]. Specifically, the mushroom industry generates a substantial supply of an organic byproduct called spent mushroom substrate (SMS), which includes the fungal mycelium and the unutilized substrate left after harvesting mushrooms. Additionally, hydroponic cultivation contributes to the production of agro-industrial waste, not only through the hydroponic waste solution [3,4], the reuse of which has been the focus of many studies, but also through the remaining roots and other plant parts. Generally, most of this waste is currently disposed of by incineration or landfilling, posing significant environmental and economic challenges and becoming a source of microorganism proliferation, which can become problematic on a larger scale [5]. However, if these residues are utilized to support food production, they will no longer be considered waste, but rather new valuable resources [6].

The production of *Pleurotus* spp., commonly referred to as ‘oyster’ mushrooms, has been increasing rapidly because of the ease of their cultivation on many lignocellulosic residues. Unlike other mushrooms, oyster mushrooms can comfortably grow on a wide range of agricultural waste due to their complex enzymatic systems, and they have attracted special attention for their high nutritional value and medicinal importance. In particular, this genus comprises about 40 species, including *P. citrinopileatus* (golden oyster mushroom), which is well known for its attractive shape and yellow color, along with the highly appealing cashew-like flavor that eventually develops when it is cooked until crispy [7]. It is recommended as a healthful food with high protein and fiber content but low levels of lipids [8,9,10], while it is also rich in vitamins B1 (thiamine), B2 (riboflavin), B3 (nicotinic acid), B5 (pantothenic acid), B6 (pyridoxine), B7 (biotin) and B9 (folic acid) [11]. Additionally, accumulating evidence has shown that water extracts from *P. citrinopileatus* have many beneficial functions, including antitumor activity [12], immune-enhancing ability [13] and antihyperglycemic properties [14]. The polysaccharides from *P. citrinopileatus* fruiting bodies are important active ingredients with various activities, such as antioxidant, hepatoprotective, anti-inflammatory, immunomodulating and anti-obesity properties [15,16,17,18]. Furthermore, Musieba et al. [19] stated that *P. citrinopileatus* mushrooms can be an excellent source of micronutrients and antioxidant components, while Rushita et al. [14] reported that *P. citrinopileatus* had excellent antidiabetic activity, highlighting its great potential as an ingredient in natural health products.

The byproducts generated in mushroom production represent a good source of valuable compounds, including polysaccharides and proteins [20]. Numerous in vivo studies have demonstrated that mushroom extracts, particularly carbohydrate extracts, can lead to the increased activity of antioxidant enzymes, including superoxide dismutase (SOD) and catalase (CAT), which are crucial components of the body’s defense system against reactive oxygen species (ROS) [21]. However, the molecular mechanisms by which mushroom polysaccharides modulate the antioxidant activity of these enzymes remain unclear. Exopolysaccharides extracted from the mushroom *P. geesteranus*, primarily composed of α-glucan, enhance the activity of SOD, GSH-Px and CAT [22]. Another study by Muszynska et al. [23] indicated that the expression of nuclear factor (erythroid-derived 2)-like 2 (NFE2L2) increased after incubation with *Agaricus bisporus* mushroom extract in colon epithelial Caco-2 cells activated by lipopolysaccharide (LPS) and tumor necrosis factor. Different mushroom species may produce distinct compounds with antioxidant activity. Additionally, a single mushroom species might produce various antioxidant compounds [24]. Mushroom polysaccharides have unique physical characteristics compared to other natural polysaccharides in terms of their molecular weights, chemical properties, degrees of branching, skeleton lengths, three-dimensional structures and other characteristics [21]. Therefore, further studies are needed to elucidate the molecular mechanisms by which mushroom polysaccharides regulate the expression profiles of antioxidant genes in mammalian models and their involvement in the Nrf2/HO-1 pathway.

Considering the potential for the commercial production of *Pleurotus* spp. mushrooms on new, low-cost substrates, which holds promise in financial and environmental terms, within the context of the circular economy, this study aimed to evaluate the performance of *P. citrinopileatus* in solid-state fermentation (SSF) by combining SMS with HRL. This evaluation involved assessing productivity and analyzing the biochemical characteristics and overall quality of *P. citrinopileatus* fruit bodies. Specifically, the study initially examined the colonization rate of the fungus in the vegetative phase, while further scale-up experiments were carried out to analyze the effects of these combined substrates on the quality and nutritional and bioactive characteristics of the mushrooms, including fruit body production and morphological characteristics; protein, carbohydrate, lipid and ash contents; fatty acid and monosaccharide composition; total phenolic content; total flavonoids; total terpenoids; and antioxidant activity.

## 2. Materials and Methods

### 2.1. Fungal Strain and Substrates’ Preparation

In the present study, a commercial strain of *P. citrinopileatus*, AMRL 155, belonging to the fungal culture collection of the Laboratory of Edible Fungi/ITAP/Hellenic Agricultural Organization—Dimitra, was examined. The cultures were maintained on Potato Dextrose Agar (PDA; Merck, Darmstadt, Germany) for routine culture and storage purposes. Before each experiment, fungal strains were reproduced in PDA Petri dishes by incubation at 26 ± 1 °C and a relative humidity of 75%. The grain spawn of *P. citrinopileatus* was prepared as previously described [25].

Spent mushroom substrate (SMS) and the hydroponic roots of leafy vegetables (HRL) were derived from large-scale experiments conducted by the Greek agricultural company Manitus S.A. and were combined in different ratios, as shown in Table 1, to create 12 new substrates. To most of them, wheat bran (WB) and soybean flour (SF) were added, whereas 1% *w*/*w* calcium carbonate (CaCO_3_; SDS, Peypin, France) was added to all substrates to obtain a pH value around 7. A substrate consisting of wheat straw (WS) was the control substrate. pH and electrical conductivity were measured by a Crison (Barcelona, Spain) GLP 21 pH meter and a Hanna HI-8733 (Padova, Italy) conductivity meter.

After soaking and drainage (moisture content of 75–80%), five glass tubes (200 × 28 mm, 80 mL volume) and three autoclavable polypropylene bags (1 kg) were filled with each substrate and autoclaved at 121 ± 1 °C for 2 h (1.1 atm). Then, tubes and bags were inoculated with *P. citrinopileatus* grain spawn under aseptic conditions. Substrate colonization took place in a growth chamber (DRAWELL, mod. D.W.-LBI-400, Shanghai, China) in the dark (24 ± 1 °C, 85% relative air humidity) until full colonization. Glass tubes were used to evaluate the mycelial growth rate of fungal colonies (Kr; mm/d) by measuring the visible penetration of mycelia into the substrate in two perpendicular directions every few days [26]. After complete colonization, the bags were transferred to a fruiting room (ENTERLAB, mod. GROW-1300 HR, Terrassa, Spain) with specific environmental conditions for fruit body induction (20 ± 1 °C, RH 90–95%) and fructification (17 ± 1 °C, RH 85%). During harvesting, the light intensity was set at 800 lux (12 h/day, fluorescent lamps), the air exchange rate was controlled to maintain a low CO_2_ level (<1000 ppm), relative air humidity was adjusted to 95% and the temperature was set at 17 ± 1 °C. The bags were used to evaluate the earliness period (elapsed time between the day of substrate inoculation and the day of the first harvest) and the biological efficiency (B.E.%; the ratio of the weight of fresh mushrooms to the d.w. of the substrate). In addition, the quality of mushrooms harvested from each substrate (obtained from the first and the second production flushes) was examined. After being frozen at T = −20 ± 0.5 °C and lyophilized (in a HetoLyoLab 3000, Heto-Holten Als, Denmark), whole fruit bodies were ground (in a Janke & Kunkel, IKA-WERK, analytical mill, Germany) to a fine powder. Two successive crops were processed. Analyses were conducted on three samples derived from the three bags per crop (six replicates).

### 2.2. Analytical Methods

The organic matter and total nitrogen in the cultivation substrates were determined according to Sparks [27] and the Kjeldahl (Total Kjeldahl Nitrogen, TKN) Method [28]. Samples of mushrooms were analyzed for chemical composition, total phenolic compounds and antioxidant activity.

The crude protein content of the samples was estimated by the Bradford method [29]. To 100 μL of the sample, 1900 μL of Coomassie Brilliant blue solution was added and the mixture was incubated at 25 ± 1 °C for 10 min and then measured photometrically at 595 nm.

Total cellular lipids were extracted from the dry biomass by a chloroform/methanol 2/1 (*v*/*v*) mixture and determined gravimetrically [30]. Fatty acids were determined by GC in a Varian CP-3800 chromatograph equipped with a flame ionization detector (Agilent Technologies, Santa Clara, CA, USA) containing an Agilent J&W Scientific DB23 capillary column (model n.123–2332, 30.0 m × 0.32 mm, film thickness 0.25 μm) (Agilent Technologies, Santa Clara, CA, USA). Helium gas was used as a carrier gas with a column flow rate of 2.0 mL/min. The set-up conditions were as follows: the initial oven temperature was set at 150 ± 1 °C, held for 18 min, subsequently ramped to 185 ± 1 °C at a rate of 5 °C/min and held for 2 min. Then, the oven temperature was raised to 210 ± 1 °C at a flow rate of 5 °C/min held for 2 min and then increased to 240 ± 1 °C at 10 °C/min. The injector and flame ionization detector temperatures were set at 260 ± 1 °C and 270 ± 1 °C, respectively. Individual fatty acid methyl-esters were identified by comparing their retention times with external standard (Supelco 37 Component fatty acid methyl-esters Mix, CRM47885, Merck KGaA, Darmstadt, Germany) retention times. The amounts of individual fatty acid methyl-esters identified are expressed in % of the total fatty acid area identified in chromatograms.

Total carbohydrates were calculated by difference, rather than analyzed directly. In this approach, the other constituents in the fruit bodies (protein, fat, water, ash) are determined individually, summed and subtracted from the total weight of the food. This is referred to as total carbohydrate by difference and is calculated by the following formula: 100 − (weight in grams [protein + fat + water + ash] in 100 g of dry fruit bodies). The composition of individual saccharides was determined by HPLC analysis. The extract was prepared as described by Diamantopoulou et al. [31,32]. Thus, filtered aliquots of the samples neutralized with NaOH were analyzed by a Waters Association 600E apparatus with a 30.0 cm × 7.8 mm column Aminex HPX-87H (Bio-Rad, Hercules, CA, USA). The mobile phase used was H_2_SO_4_ at 0.005 M with a flow rate of 0.8 mL min^−1^, and the column temperature was 65± 0.5 °C. Individual simple sugars and sugar alcohols were detected by an RI detector (differential refractometer 410-Waters).

Methanolic extracts were prepared as follows: 250 mg of fresh mushrooms were extracted with 5 mL of methanol in an ultrasonic bath (SKYMEN, JP-060S, Shenzhen, China) for 60 min at 25 ± 0.5 °C, followed by vortexing and centrifugation (3500 rpm, 15 min, 25 ± 0.5 °C; Micro 22R, Hettich, Germany). The extraction was repeated three times and the supernatants were stored at 4.0 ± 0.5 °C for further analysis. The total phenolic compounds (TPCs) in mushroom samples were estimated using the Folin–Ciocalteu (FC) assay by measuring the absorbance at 750 nm [33]. Free radical scavenging activity was determined according to Re et al. [34] with some modifications. The ability to scavenge DPPH˙ free radicals was determined according to Boonsong et al. [35]. The sample’s capacity to convert Fe^3+^ to Fe^2+^ ions is the basis for the method described by Benzie et al. [36] and the results are expressed as mmol trolox equivalents per 100 g of dry matter. Total flavonoid content (TFC) was determined by the colorimetric method described by Barreira et al. [37], with some modifications and is expressed as milligrams of quercetin equivalents (mg quercetin/g d.w.). Total triterpene (Tr) content was evaluated according to Fan and He [38], with some modifications and the results are expressed as mg ursolic acid equivalents (mg UA equivalent).

For the alkaline solubilization of proteins and precipitation at the isoelectric point, the amount of powder specified for each mushroom sample was dissolved in deionized water at a 1:10 ratio [39]. Using a pH meter and 2.0 M NaOH solution, the pH of the mushroom mixtures was adjusted to 11–12, a value at which proteins exhibit maximum solubility according to the literature. The mixtures were centrifuged at 6000 rpm at 4 °C for 20 min. After centrifugation, the supernatant was collected and the pH was adjusted to 4, the isoelectric point of the protein, using a 2.0 M HCl solution. The resulting mixtures were centrifuged at 6000 rpm at 4 °C for 20 min. The supernatant from each tube was discarded and the precipitates were placed in a freezer for 24 h and then dehydrated using a freeze-drying device for approximately 24 h [40]. The dry concentrate obtained was weighed.

To recover lipophilic compounds, 10 mL of ethanol was added to 1 g of sample (dry lyophilized biomass), followed by stirring for 24 h. The sample was then centrifuged (6000 rpm/10 min/4 ± 0.5 °C) and the biomass was recovered and dehydrated through drying. Cell disruption to release the intracellular molecules was carried out by using an ultrasonic water bath for 1 h at 80 ± 1 °C. Subsequently, centrifugation (6000 rpm/10 min/4 °C) was performed and the supernatant liquid was recovered and stored at 4 ± 0.5 °C. The removal of water-soluble macromolecules of a non-polysaccharide nature was achieved using the Sevag method (chloroform/n-butanol, 5:1) [41]. For each volume of the supernatant liquid, half the volume of the Sevag mixture was added (extract/Sevag, 2:1). This was followed by centrifugation (9000 rpm/10 min/4 ± 0.5 °C) and the upper phase was collected. The process was repeated until there were no more proteins. Protein detection was performed using the Bradford method [29] to verify the removal of proteins. The final supernatant was collected and polysaccharides were precipitated with ethanol at a 1:4 ratio at 4 ± 0.5 °C. Finally, the polysaccharides were isolated by centrifugation and the precipitates were placed in a freezer for 24 h, followed by freeze-drying dehydration.

### 2.3. In Vitro Digestion Protocol and Digests’ Fractionation

The in vitro digestion protocol followed the detailed methodology outlined by Dalaka et al. [42]. This procedure was based on the harmonized INFOGEST 2.0 in vitro digestion protocol, designed to mimic the conditions in the oral, gastric and intestinal phases [43,44]. The experimental procedure was conducted separately for protein extracts (PEs) and carbohydrate extracts (CEs) from six different samples of *P. citrinopileatus*. Samples (5 mL) were prepared by dissolving the extracts in Mili Q water (Merck Millipore, Burlington, MA, USA) to final concentrations of 40 mg/mL PE and 2.25 mg/mL CE. These were stored at 4 °C overnight to ensure complete rehydration. In more detail, the following formulations were used for both extracts: SMS 80%, SMS 80%-HRL 10%, SMS 80%-HRL 20%, SMS 60%-HRL 30%, SMS 60%-HRL 40% and WS (control substrate). As salivary amylase is needed only to digest starch-containing food [44], 0.17 mL (E-BLAAM enzyme activity 3000 U/mL) of α-amylase was added only to CE samples for 2 min at pH 7 during the oral phase [45]. Following the completion of the intestinal digestion phase, the PE and CE digests (PE-Ds and CE-Ds) were immediately heated at 85 °C for 10 min and then placed on ice. Samples were centrifuged and the supernatants were filtered through 0.22 μm sterile PVDF syringe filters. To obtain fractions between 0 and 3 kDa (PE-D-P3), membrane filters (Ultracel^®^ low binding regenerated cellulose) (Merck Millipore, Burlington, MA, USA) with a molecular weight cut-off (MWCO) of 3 kDa were used. Afterward, the samples were stored at −20 °C until subsequent analyses. Digestion was performed for all samples (PEs and CEs) in duplicate. In parallel, four replicates of blank digests were also prepared for each experiment using water instead of a sample, following the same process of the in vitro digestion protocol. The corresponding fractions after in vitro digestion were named BL-D for the blank digest and BL-D-P3 for the fraction of digestate containing peptides with a molecular weight below 3 kDa.

### 2.4. Cell Culture and Activation of THP-1

Cells from the human acute monocytic leukemia cell line THP-1 were cultured in RPMI 1640 medium, supplemented with 10% (*v*/*v*) fetal bovine serum, 10 U/mL L-glutamine, 1 mM sodium pyruvate, 100 U/mL penicillin, 100 μg/mL streptomycin and 100 μM non-essential amino acids in a humidified incubator at 37 °C and 5% CO_2_. Monocytes were seeded in 6-well plates (2.5 mL/well) at a density of 0.8 × 10^6^ cells/mL and treated with 100 ng/mL PMA for 48 h. After this period, the PMA-containing medium was removed and the cells were washed with PBS and then incubated in PMA-free supplemented RPMI 1640 medium for an additional 24 h. Following this resting phase, macrophages were exposed to 100 ng/mL lipopolysaccharide and either PE-D-P3 (10%) for 24 h or CE-D (15%) for 6 h. In parallel, BL-D-P3 and BL-D were used, respectively. Each sample was analyzed in triplicate.

### 2.5. Quantification of Gene Expression in LPS-Stimulated Macrophage THP-1 Cells

Total RNA was extracted from the attached THP-1 cells using the Nucleozol reagent (Macherey-Nagel, Duren, Germany) following the manufacturer’s protocol. To remove DNA traces, samples were treated with DNase I. RNA quantity and purity were assessed with a Q5000 spectrophotometer (Quawell Technology Inc., San Jose, CA, USA). Subsequently, reverse transcription was performed. Briefly, 500 ng of total RNA was processed using PrimeScript RT (Takara Bio, Shiga, Japan) according to the manufacturer’s instructions. Quantitative PCR (qPCR) was conducted with a thermal cycler (SaCycle96, Sacace Biotechnologies, Como, Italy) utilizing FastGene 2 × IC Green qPCR Universal Mix (Nippon Genetics, Tokyo, Japan). Primers for the target genes (*NFE2L2*, *SOD1*, *CAT*, *HMOX1* and *GSR*) and housekeeping genes (*B2M*, *RPS18* and *RPL37A*) were designed with a 60 °C annealing temperature. Each qPCR reaction was performed in duplicate. Relative gene expression was calculated using a modified version of the Pfaffl method against the aforementioned housekeeping genes [46]. Primer details are provided in Table 2.

### 2.6. Statistical Analysis

Variance analysis was performed using the Least Significant Difference (LSD) test at a 5% level of probability to compare the mean values of the parameters tested. All experimental data presented in Section 3.5 are reported as means ± standard error of the means (SEMs) from a minimum of two biological replicates. Normality was assessed using the Kolmogorov–Smirnov test and data were transformed logarithmically or normalized [47] when necessary to achieve a normal distribution. Subsequently, one-way ANOVA was conducted, followed by Duncan’s post hoc test. Differences between means were deemed significant at *p* < 0.05. All statistical analyses were carried out using SPSS for Windows, version 22.0.0. Data visualization in Section 3.5 was performed with GraphPad Prism 8.

## 3. Results and Discussion

### 3.1. Evaluation of Different Substrates for P. citrinopileatus Cultivation

The final mixtures of SMS and HRL were prepared based on the ratio of C/N (15–36). A variety of C/N ratios have been reported in previous studies for cultivation substrates of *P. citrinopileatus* [48]. The total nitrogen content in the substrates SMS 100% and SMS 90%-HRL 10% were below 1% and consequently, the final C/N ratio was increased. The pH values were also suitable for mushroom cultivation, while the EC values significantly varied among substrates.

**Figure 1:**
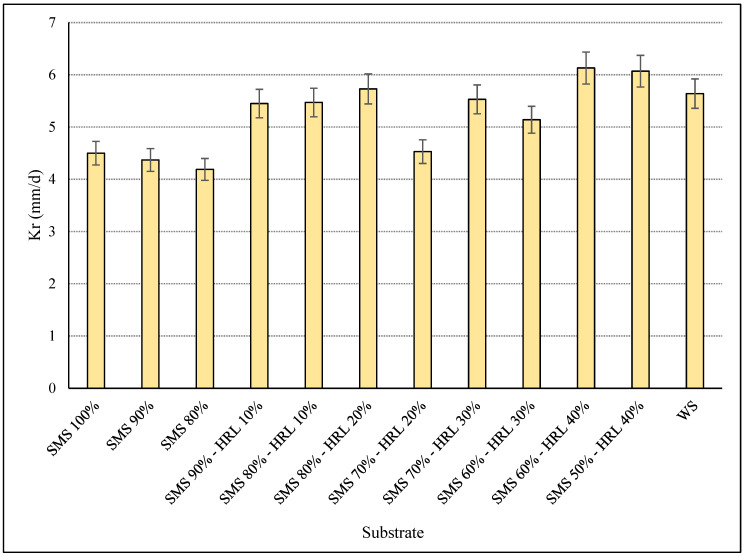
Growth rates (Kr, mm/d) of *P. citrinopileatus* during solid-state fermentation in glass tubes on substrates consisting of spent mushroom substrate (SMS) and the hydroponic roots of leafy vegetables (HRL) (control substrate: wheat straw—WS). Mean values with error bars indicate the standard deviations (from duplicate experiments) of six replicates.

The effect of different substrates consisting of SMS or a combination of SMS and HRL, supplemented or not with additives, on *P. citrinopileatus* growth was initially examined through the mycelial growth rate (Kr, mm/d). In general, HRL addition led to a faster Kr than the substrates only consisting of SMS (Figure 1). Specifically, the highest Kr value (6.1 mm/d) for *P. citrinopileatus* was recorded on substrates with the highest ratio of HRL (SMS 60%-HRL 40% and SMS 50%-HRL 40%), higher values than the control substrate (WS). Regarding substrates with only SMS, the mycelial growth rate of *P. citrinopileatus* increased progressively with the SMS increase, as has already been reported by Wang et al. [49]. Also, it is noteworthy that the lack of additives in WB and SF did not negatively affect Kr values. In previous studies, faster growth rates (6–8 mm/d) were detected for *P. citrinopileatus* cultivated on substrates consisting of the stalks of several grass plants [48], whereas *P. ostreatus* and *P. eryngii* achieved lower Kr values (3.1 and 2.6 mm/d, respectively) on commercial SMS [50] than samples in the present study. As combinations of different ratios of SMS and HRL have not been examined as alternative substrates for new mushroom cultivations, in the subsequent larger-scale experiments (1 kg bags), all the above substrates were tested. 

*P. citrinopileatus* successfully grew and colonized all of the alternative substrates used in this study within a period of 17 to 20 days after inoculation. The earliness period ranged from 26 to 30 days and in most cases, HRL supplementation favored a decrease in earliness (Table 3). In previous studies, further SMS supplementation had a positive role in reducing the total *P. ostreatus*, *P. eryngii* and *P. pulmonarius* cultivation time [50,51]. However, this trend was not observed in the present study for *P. citrinopileatus*; the growing period of mushrooms depends on many parameters, such as the type of strain, the selected lignocellulosic cultivation substrate, the biological structure of the substrate and the additive ratio. A similar earliness period (26–30 days) was also detected by Kulshreshtha et al. [52] when they cultivated *P. citrinopileatus* on substrates consisting of WS with cardboard industrial waste and WS with handmade paper industrial waste (1:1 *w*/*w*), while a longer earliness period occurred on the control substrate, consisting of 100% WS (38.6 days). Atila [53] recorded a longer earliness period for *P. citrinopileatus* cultivated on oak sawdust, safflower hay, bean straw and sunflower head residue from 38 to 46 days.

Regarding the biological efficiency (B.E., %), although all values were lower than that on the control substrate, satisfactory values (up to 50%) were detected on substrates consisting of 70–80% SMS and 10–20% HRL (Table 3). The highest B.E. values were recorded on SMS 70%-HRL 20% (61.0%) among the alternative substrates. On the other hand, the highest ratio of HRL led to the lowest B.E. (39.2%), while the B.E. was increased when the other substrate with the highest ratio of HRL supplemented with additives (SMS 50%-HRL 40%) was used. As has already been mentioned, substrate supplementation with protein-rich additives (wheat bran, soybean flour) increases mushroom B.E. [51,54]. In the present study, substrate supplementation led to higher B.E. values than the corresponding substrate without additives. For example, the B.E. value was 41.9% on SMS 90%-HRL 10% and increased to 52.7% after additive supplementation in the cultivation substrate SMS 80%-HRL 10%. However, one of the highest B.E. values, 55.9%, was recorded on SMS 80%-HRL 20%, a substrate without additives, indicating that there are crucial physicochemical parameters other than additives that can affect mushroom B.E., such as the availability of nutrients, the moisture content, the cellulose and lignin contents, the pH and the EC of the cultivation substrate. In a previous study, higher B.E. was recorded for *P. eryngii* cultivated on a substrate consisting of SMS and *Pleurotus* waste (stipes, mishappen mushrooms) without additives (97.97%) than on the same one with additives (87.80%) [50].

Similar B.E. values were detected for *P. citrinopileatus* in various grass plants, from 40 to 65% [48] and in different agro-residues, from 42.5 to 73.9% [53]. Kulshreshtha et al. [52] and Koutrotsios et al. [55] reported lower B.E. values for *P. citrinopileatus* cultivated on WS: 58.2% and 53.7%, respectively. Higher values (88.9–94.5%) were recorded in mixtures of handmade paper and cardboard industrial waste with wheat straw [52] and in grape marc mixed with wheat straw (78.5%), whereas *P. citrinopileatus* B.E. was not favored in olive mill byproducts (26.2%) [55].

The results of this study revealed the successful bioconversion of alternative substrates, mixtures of SMS and HRL, by *P. citrinopileatus*, which has not been examined before. In particular, the mushroom grew very well on the SMS 80% substrate and on mixtures of SMS with the lowest supplementation of HRL (SMS 80%-HRL 10%, SMS 80%-HRL 20% and SMS 70%-HRL 30%), indicating that SSF is a possible solution to deal with the mushroom industry’s byproducts and agro-industrial waste.

Concerning the quality of the fruit bodies formed, the average pileus diameter and stipe length of *P. citrinopileatus* fruit bodies ranged from 3.7 to 5.2 cm and from 1.9 to 3.2 cm, respectively (Table 3). In general, no significant differences were noticed in the appearance of *P. citrinopileatus* fruit bodies cultivated on different substrates, as has already been mentioned in previous studies [55,56]. Jatily et al. [57] reported a slightly higher stipe length (3.5 cm) and bigger pileus width (7.0 cm) for *P. citrinopileatus* cultivated on wheat straw. So, the alternative substrates used in the present study can be utilized to produce high-quality *P. citrinopileatus* fruit bodies with commercially desirable morphological characteristics [58].

### 3.2. Nutrient Composition of Fruit Bodies/Macronutrients of P. citrinopileatus

The fruit bodies of mushrooms are rich in carbohydrates, proteins and minerals, with very low fat content. Chemical constituents were estimated for fruit bodies produced from the first and the second flushes. The composition reported for *P. citrinopileatus* cultivated on different substrates showed that the protein content of fruit bodies from the alternative substrates was significantly higher (25.6 to 34.7% d.w.) compared to the control substrate (24.4% d.w.) (Table 4). In this study, replacing WS with SMS and adding HRL, which contains more nitrogen, appears to have positively affected the mushroom protein content. Nitrogen is a crucial nutrient for fungal growth, serving as a vital component of amino acids, the building blocks of proteins. So, by increasing the nitrogen content of the substrate, mushroom growers could enhance the protein content of the fruit bodies. This improvement could have a positive impact on the nutritional value and marketability of the mushrooms.

The lipid content was generally low, ranging from 2.6 to 5.1% d.w., in *P. citrinopileatus* fruit bodies grown on different substrates. Also, negligible differences were detected in the ash content of *P. citrinopileatus,* regardless of the cultivation substrate, with values ranging from 6.7 to 8.4%. The total carbohydrate values ranged between 52.0 and 65.7%, which are considerably high. However, the carbohydrate content estimated by difference includes fiber, as well as some components that are not strictly carbohydrates, e.g., organic acids [59].

These findings are supported and confirmed by many authors. Singh et al. [60] reported higher protein content, above 40% and lower total carbohydrates for *P. citrinopileatus* cultivated on vegetable waste. Musieba et al. [19] reported that *P. citrinopileatus* contained 22.1% protein, 1.3% crude lipid and 20.78% fiber. Many studies reported that *P. citrinopileatus* contained (on a d.w. basis) 16–25% protein, 19–28% carbohydrate and about 9% crude fiber [61,62]. Another study by Ahmed et al. [63] revealed that *Pleurotus* species contain 86–90% moisture, 28–31.8% protein, 3.5–4.7% fat and 8.6–12.8% ash on a dry weight basis. Ragunathan [64] found that mushrooms grown on various agro-residues have a nutrient composition of 41.5–44.4% carbohydrates, 30.1–40.6% proteins and 1.7–2.9% lipids. Overall, *P. citrinopileatus* mushrooms emerge as excellent sources of essential nutrients, reaffirming their potential as valuable functional foods.

### 3.3. TPC, TFC, Scavenging Activity of DPPH Radical, Scavenging Activity of ABTS Radical and Ferric Reducing Antioxidant Power (FRAP˙) in Methanol Extracts

**Figure 2:**
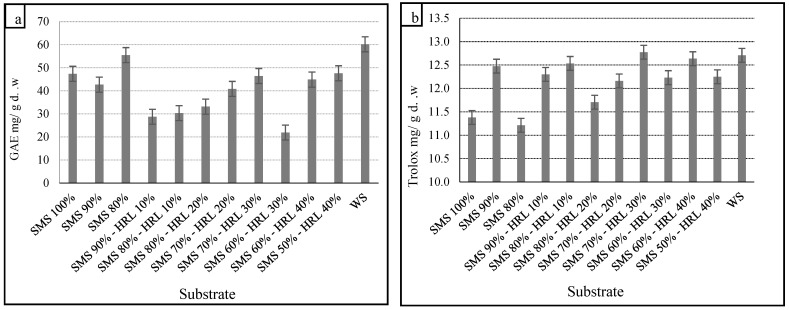

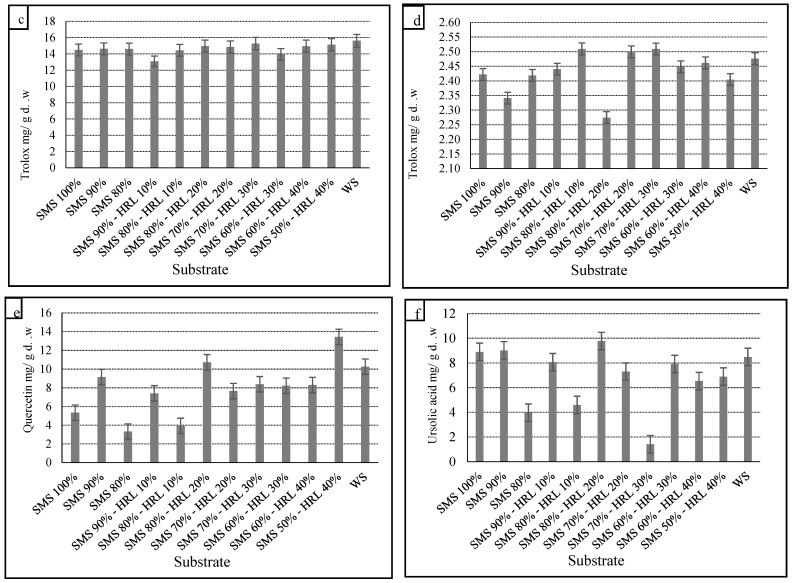
(**a**) Total phenolic compounds (TPCs, expressed as gallic acid equivalents, GAE mg/g d.w. of biomass) (**b**) DPPH scavenging activity, (**c**) scavenging of radical ABTS^•+^, (**d**) ferric reduction activity power (FRAP) expressed in mg Trolox/g d.w., (**e**) total flavonoid content (TFC) expressed in mg quercetin/g d.w. and (**f**) total triterpene (Tr) expressed as mg ursolic acid equivalents of the extracts of *P. citrinopileatus* grown on substrates consisting of spent mushroom substrate (SMS), the hydroponic roots of leafy vegetables (HRL) (control substrate: wheat straw—WS). Mean values with error bars indicate the standard deviations (from duplicate experiments) of six replicates.

Edible mushrooms are known to be a source of phenolic compounds with antioxidant properties [65]. Our results indicated significant differences in TPCs in the methanol extracts of the mushrooms. Previous studies have reported the impact of the substrate’s chemical composition on the TPCs in mushrooms [66,67]. The highest TPC was 60.2 mg GAE/g d.w. on the control substrate, while significantly lower TPC was found on the alternative substrates, with the minimum concentration being 21.9 mg GAE/g d.w. on SMS 60%-HRL 30%. The TPCs in mushroom extracts are shown in Figure 2a.

The presence of phenolic compounds is an indication of antioxidant activity. Research has shown a strong correlation between antioxidant activity and phenolic content [68]. Mushroom extracts contain significant quantities of phenolic compounds, which include aromatic rings with hydroxyl groups. These compounds act as free radical scavengers, hydrogen donors, electron donors and metal ion chelators. More hydroxyl groups in phenolics may enhance antioxidant activity [69]. Most studies show that the DPPH scavenging activities of antioxidants depend on their hydrogen-donating abilities [70]. Figure 2b demonstrates DPPH scavenging activity, expressed in mg Trolox/g d.w. The potential of mushrooms to scavenge ABTS radicals ranged from 2.3 to 2.5 mg Trolox/g d.w. (Figure 2c). Antioxidant activity was also measured using the FRAP method and was observed in all extracts (Figure 2d). The antioxidant activity of extracts of the dried samples ranged from 13.1 to 15.6 mg Trolox/g d.w. Lee et al. [71] measured values of 2.3, 15.2 and 16.5 mg/mL for ethanolic, cold water and hot water extracts, respectively, in *P. citrinopileatus* fruit bodies. Aqueous extracts of *P. citrinopileatus* grown on a substrate mix (38% *w*/*w* sawdust) showed the highest FRAP values (2.4 mg EAA/g extract) and ABTS radical scavenging activity (7.1 mg EAA/g extract) [63]. The potential of *P. citrinopileatus* to scavenge ABTS radicals ranged from 0.6 to 14.1 mg Trolox/g d.w. for the water extract, while the ethanol extract ranged from 0.4 to 11.8 mg Trolox/g d.w. [72]. Despite the high antioxidant potential described by Freitas et al. [63] and Lee et al. [71], different sample processing methods and result expressions do not allow direct comparisons.

The TFC in all samples was low, ranging from 3.4 to 13.4 mg QE/g (Figure 2e). Flavonoids represented a very small percentage of TPCs. Arbaayah and Umi [73] reported high flavonoid contents in ethanol extracts of various *Pleurotus* species, with values of 1.4 to 29.8 mg QE/g. Jan-Ying et al. [74] reported the flavonoid content of ethanolic, cold water and hot water extracts of two *Grifola frondosa* strains, with values of 1.1–3.1 and 0.1–0.8 mg QE/g dry sample, respectively. Sudha et al. [75] reported the flavonoid content of ethyl acetate, methanol and hot water extracts of *Pleurotus eous*, with values of 6.4 to 7.8 mg catechin equivalents (CAE)/g extract. However, Gil-Ramírez et al. [76] reported that mushrooms do not contain flavonoids and those found in hyphae may be due to their ability to absorb nutrients and compounds from substrates or neighboring plants by spreading their hyphae or forming mycorrhizae. Some plants release flavonoids to regulate symbiotic plant–microbe interactions, defining the species that can tolerate growth on their roots. Flavonoids are reported to be antifungal compounds produced by plants to protect against fungal infections, which might negatively affect fungal growth.

The Tr content in sample ethanol extracts (mg/g extract) is presented in Figure 2f. The content per d.w. (mg/g d.w.) varied depending on the substrate, with the maximum Tr value detected in SMS 80%-HRL 20% (9.8 mg UA/g d.w.). According to researchers [77,78], triterpenoids and polysaccharides are valuable biologically active substances in mushrooms. Li et al. [79] reported that the d.w. of total triterpenoid content in *G. lucidum* fruit bodies ranged from 13.6 to 31.5 mg/g, higher than our results. Boh et al. [80] found that the total triterpenoid content was higher at younger growth stages of Indonesian *Ganoderma applanatum* than at older stages. Feng et al. [81] also demonstrated that the total triterpenoid content at the immature stage was much higher than at the mature stages. In conclusion, edible mushrooms are rich sources of phenolic compounds, which exhibit significant antioxidant properties.

### 3.4. Fatty Acid (FA) Composition and Individual Saccharide Profile

From the data presented in Table 5, it was found that the PUFA concentration ranged from 71.2 to 82.5%, *w*/*w*, whereas saturated FAs varied from 0.2 to 13.9%, *w*/*w*, significantly lower concentrations. This agrees with many previous observations that unsaturated FAs predominate over saturated ones in mushroom mycelia and fruit bodies [31,82]. All samples showed high linoleic acid (C18:2) concentrations, above 60 g FA/100 gfat, indicating that this is the main FA in the composition of *P. citrinopileatus* (63.8–75.2 g FA/100 g fd.w.). The maximum concentration of linoleic acid was detected in fruit bodies cultivated on SMS 60%-HRL 30%. The FA composition of the mushroom species was characterized by much higher contents of mono- and polyunsaturated FAs than saturated FAs. The saturated FA distribution was characterized by palmitic (C16:0) and stearic acids (C18:0). A higher content of palmitic acid was measured in *P. citrinopileatus* (10.8–13.9 g FA/100 g d.w.). Interestingly, the concentration of linoleic acid was higher than 63%, but the concentration of oleic acid (C18:1) was the lowest (4.6–9.4 g FA/100 gfat). The FA composition of edible mushrooms contains a wide range of FAs, from lauric acid (C12:0) to docosahexaenoic acid (C22:6), depending on the mushroom species. However, the main FAs typically found are C16:0, C18:1 and C18:2 [31,82,83]. Previous studies on *P. citrinopileatus* have reported similar observations regarding fatty acid composition. Linoleic acid (C18:2) was the most abundant FA in mushrooms, ranging from 54.5 to 70.5% of total FAs, followed by palmitic acid (C16:0; 12.3–14.3%) and oleic acid (C18:1; 3.9–4.9%) [55]. Rodrigues et al. [10] reported higher levels of palmitic acid (16.5%) and similar levels of oleic acid (5.5%), while Reis et al. [84,85] found that the saturated FAs ranged from 17.4 to 25.8%, the monounsaturated fatty acids ranged from 13 to 49% and the PUFAs ranged from 25.2 to 69.4% in wild *Pleurotus* spp. To our knowledge, this is the first study describing the FA composition of the edible *P. citrinopileatus*; therefore, this work provides valuable data on the nutritional evaluation of such mushroom species.

The carbohydrate composition analysis results are summarized in Table 6. Fructose was identified as the primary constituent in *P. citrinopileatus* fruit bodies when the substrate mix contained less than 80% SMS (up to 70%, *w*/*w*). Glucose was presented in smaller quantities, around 52%, *w*/*w*, while mannose was detected in lower concentrations or not at all in some samples. Fructose, commonly known as fruit sugar, is distinctly sweeter than glucose and sucrose, with its perceived sweetness varying based on its concentration and the presence of other compounds. It is essential to consume fructose in moderation as part of a balanced diet [86,87]. Generally, differences in sugar composition have been observed between cultivated and wild samples of the same mushroom species, likely due to different cultivation techniques [88].

### 3.5. Effect of Protein and Carbohydrate Extracts on Expression of Antioxidant Genes

This study is the first to present substantial evidence regarding the antioxidant-related gene expression induced by the protein extract (PE-D-P3) and polysaccharide extract (CE-D) of *P. citrinopileatus* following in vitro gastrointestinal digestion. Firstly, the cell viability of both groups of mushroom extracts was tested using activated THP-1 macrophages. A PE-D-P3 concentration of 4 mg/mL (10% PE-D-P3 and 90% RPMI) and a CE-D concentration of 0.34 mg/mL (15% CE-D and 85% RPMI) were found to be nontoxic to THP-1 cells and consequently, these concentrations were selected.

**Figure 3:**
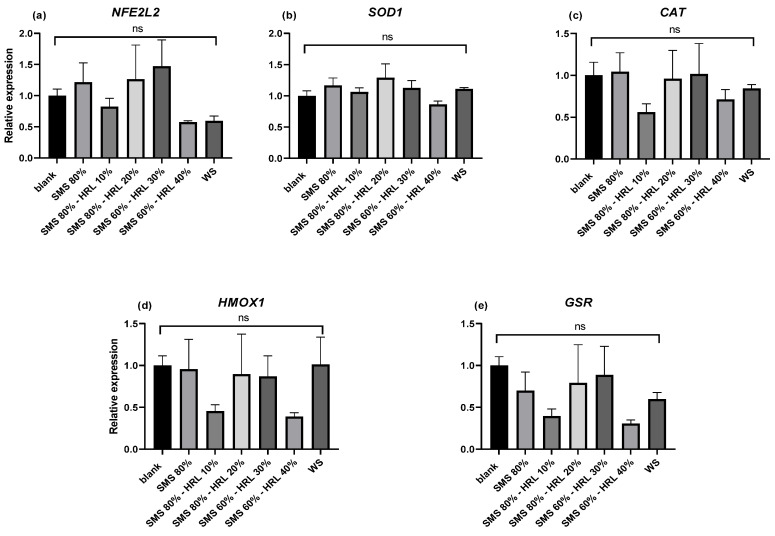
The effect of PE-D-P3 on LPS-induced mRNA expression in THP-1 cells. THP-1 cells were pretreated with PMA for 48 h (100 ng/mL), followed by a 24 h rest and then treatment with LPS (100 ng/mL) with or without the presence of 4 mg protein/mL of PE-D-P3 or BL-D-P3 (blank) for 24 h. The expression levels of (**a**) *NFE2L2*, (**b**) *SOD1*, (**c**) *CAT*, (**d**) *HMOX1* and (**e**) *GSR* were measured using qPCR and were normalized to three housekeeping genes (*B2M*, *RPS18* and *RPL37A*). Data are represented as means ± SEM; ns = not significant (*p* > 0.05).

**Figure 4:**
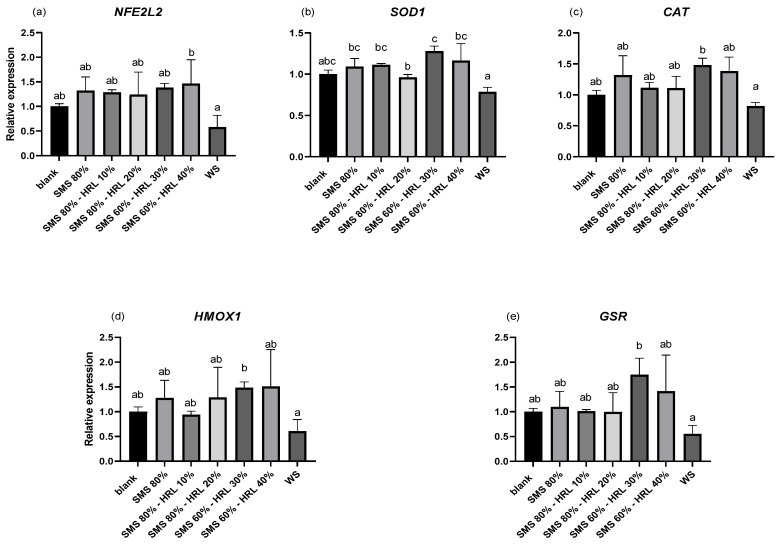
Effect of CE-D on LPS-induced mRNA expression in THP-1 cells. THP-1 cells were pretreated with PMA for 48 h (100 ng/mL), followed by a 24 h rest and then treatment with LPS (100 ng/mL) with or without the presence of 0.34 mg protein/mL of CE-D or BL-D (blank) for 6 h. The expression levels of (**a**) *NFE2L2*, (**b**) *SOD1*, (**c**) *CAT*, (**d**) *HMOX1* and (**e**) *GSR* were measured using qPCR and were normalized to three housekeeping genes (*B2M, RPS18* and *RPL37A*). Data are represented as means ± SEM. Columns with different letters within the same panel are significantly different (*p* < 0.05).

The relative gene expression of key components in antioxidant signaling pathways in response to treatment with digested protein and carbohydrate extracts (PE-D-P3 and CE-D, respectively) was assessed in LPS-challenged THP-1-derived macrophages. Figure 3 and Figure 4 show the quantification of transcription levels of a panel of genes associated with antioxidant activity. As shown in Figure 3, no statistically significant differences were observed in the PE-D-P3 samples either between different substrates or compared to BL-D-P3-treated cells regarding *NFE2L2*, *SOD1*, *CAT*, *HMOX1* and *GSR* expression (*p* > 0.05). However, CE-D treatments significantly (*p* < 0.05) altered the expression of some of the genes mentioned above. Specifically, *NFE2L2* expression was found to be higher (*p* < 0.05) in SMS 60%-HRL 40%-treated cells when compared to WS (control). No increase, however, was observed in the other SMS groups, with or without HRL, when compared to BL-D and WS (*p* > 0.05; Figure 4a). *SOD1* expression, in turn, was higher (*p* < 0.05; Figure 4b) in all SMS groups, with or without the addition of HRL, when compared to WS, but no statistical difference was observed compared to BL-D. On the other hand, exposing THP-1 macrophages to a CE cultivated on a specific substrate, a combination of 60% SMS and 30% HRL, increased the gene expression of *CAT*, *HMOX1* and *GSR* in activated THP-1 macrophages compared to the WS group. No significant differences were observed between any of the CE digests and BL-D (*p* > 0.05; Figure 4c, Figure 4d and Figure 4e, respectively).

*Pleurotus* sp. consists of a plethora of bioactive compounds, including proteins, polysaccharides like α- and β-glucans, peptides and dietary fiber, among others. These components may offer potential functional and nutritional benefits [89]. Additionally, fungal polysaccharides have been shown to have a positive effect on human health [23], with the antioxidant activity of polysaccharides in vivo typically associated with the enhanced activities of oxidative enzymes, including catalase and superoxide dismutase [90].

The ‘nuclear factor erythroid 2-related factor/heme oxygenase-1’ (Nrf2/HO-1) pathway has recently emerged as a valuable target, acting as a primary cellular sensor of oxidative stress. Nowadays, scientists are actively seeking novel Nrf2 inducers from natural sources [91]. After its nuclear translocation, Nrf2 binds to antioxidant-responsive elements (AREs), stimulating the transcription of numerous cytoprotective genes. This enhances antioxidant enzymes like SOD and CAT, as well as inducible protective genes such as HO-1. In parallel, Nrf2 is crucial for activating target genes, including HO-1, which is the only highly inducible isoform and a key enzyme in regulating heme catabolism. This process reduces cellular ROS release and promotes various cellular defense mechanisms [92]. Moreover, evidence suggests that increased Nrf2/HO-1 activity can directly downregulate the nuclear factor-kappa B (NF-κB) pathway, reducing a range of inflammatory responses and demonstrating significant anti-inflammatory properties [93]. Thus, positively influencing the Nrf2/HO-1 pathway is expected to serve dual purposes. This study indicates that using different substrates, such as SMS or a combination of SMS and HRL, can enhance Nrf2 signaling and the expression of related genes. This research highlights the link between increased nitrogen in the substrate due to HRL addition and the upregulation of the antioxidant genes mentioned only for D-CE. However, there are limited studies on digested food, particularly mushrooms and their impact on the expression of genes like *NFE2L2*, *SOD1*, *CAT*, *HMOX1* and *GSR*, which are implicated in the oxidative response in macrophage-like THP1 cells. Some mushroom substances with antioxidant properties act as inducers or cell signals, leading to changes in gene expression that activate enzymes to eliminate ROS [94].

Antioxidative enzymes, including SOD, CAT and glutathione (GPx), serve as the first line of defense against ROS in organisms [95]. Xiaoping et al. [96] showed that *G. lucidum* polysaccharides could significantly enhance antioxidant enzyme activities (SOD, CAT and GPx) in rats with cervical cancer. Chuang et al. [97] evaluated waste mushroom compost, the main byproduct of the cultivation of *Pleurotus eryngii*, as a feed supplement for broilers and concluded that all antioxidant-related mRNA levels, including *Nrf2*, *HMOX1* and *SOD1*, increased significantly following the reuse of mushroom waste compost compared to the control group. Similarly, another study [98] evaluated the effect of dietary supplementation with *Antrodia cinnamomea* mycelia powder on the mRNA expression levels of selected antioxidant genes, particularly those regulated by *NFE2L2*. This evaluation was conducted by isolating cells from broilers after they had digested and absorbed the supplemented feed and were then exposed to an LPS challenge. It is noteworthy that the expression of antioxidant genes, including *HMOX1*, *SOD1* and *NFE2L2*, was significantly elevated compared to the control group. Li et al. [24] evaluated the antioxidant effects of a polysaccharide–peptide complex from *P. abalonus* fruiting bodies and observed that the administration of this extract to aged mice had enhanced the activities of CAT and SOD enzymes with the concurrent upregulation of gene transcripts of *SOD1* and *CAT* compared to the control group. Also, a thorough review by Kozarski et al. [94] covers the antioxidants found in edible mushrooms and shows that a small number of studies have studied the *Pleurotus* genus in this context. Still, there have been no studies on the evaluation of different substrates and their possible link to genes related to antioxidant activity in vitro.

## 4. Conclusions

The present findings suggest that the SMS of *Pleurotus* spp. can be utilized alone or in blends with HRL as an alternative substrate to produce *P. citrinopileatus* fruit bodies of high quality and nutritional value. In general, HRL addition led to a faster Kr than the substrates only consisting of SMS and favored a decrease in earliness. Regarding B.E., the highest values were recorded on SMS 70%-HRL 20% (61.0%), while the highest ratio of HRL led to the lowest B.E. (39.2%). Although the B.E. values of the new substrates were lower than those of the control substrate (73.5%), all of the produced fruit bodies had higher nutritional value than those produced on the control substrate. More specifically, all alternative substrates had a positive impact on the protein content of *P. citrinopileatus* fruit bodies (25.60–34.66% vs. 24.40% for the control). Moreover, the further addition of HRL led to the production of fruit bodies that had lower carbohydrate content but were rich in fructose, whose metabolism does not require insulin and has a low impact on blood glucose levels. Although the inclusion of HRL resulted in higher lipid content than other substrates, the lipid profile was characterized by higher concentrations of mono- and polyunsaturated fatty acids (FAs) than saturated FAs. It can also be concluded that, out of the twelve substrates studied, the control substrate produced mushrooms with significantly higher amounts of total phenolics. However, the utilization of HRL in combination with SMS enhanced DPPH and ABTS scavenging activities. The FRAP values were similar across all substrates tested. In general, high flavonoid content was obtained from substrates with an increased percentage of HRL, while the triterpenoid content was enhanced mainly with the increment in SMS. Overall, the reuse of SMS and HRL residues could lead to a decrease in mushroom cultivation costs and improve environmental protection. In addition, these new alternative substrates could not only be used for the successful cultivation of *P. citrinopileatus* mushrooms but also present an opportunity to enhance the nutritional and medicinal properties of the cultivated mushrooms, thereby confirming their value as functional foods. Also, the digested protein extract had no effect on the expression of antioxidant genes in LPS-challenged THP-1-derived macrophages across all tested substrates. However, the addition of HRL led to the upregulation of NFE2L2, SOD1, CAT, HMOX1 and GSR, key components of antioxidant signaling pathways, in the CE-D samples compared to WS. This study showed that CE-D exerts an antioxidant effect in a macrophage cell line, with higher values observed in samples containing 30% and/or 40% HRL. These findings could be further validated through in vivo animal studies or human clinical trials.

## Figures and Tables

**Table 1:** The compositions of substrates consisting of spent mushroom substrate (SMS), the hydroponic roots of leafy vegetables (HRL) and the control substrate (wheat straw—WS) used for the solid-state fermentation experiments (final mixtures, before inoculation) and their physicochemical profiles.

Substrate	Substrate Composition (%, *w*/*w*)	C (% d.w.)	N (% d.w.)	C/N	pH	EC (μS/cm)
SMS 100%	SMS 100%	27.2 ± 0.5 *	0.8 ± 0.1	35.2 ± 1.3	6.8 ± 0.0	475 ± 1.4
SMS 90%	SMS 90%	29.5 ± 0.3	1.1 ± 0.3	27.8 ± 2.1	5.9 ± 0.7	655 ± 57.3
	WB 5%					
	SF 5%					
SMS 80%	SMS 80%	29.9 ± 0.6	1.4 ± 0.2	23.4 ± 2.0	5.8 ± 0.8	680 ± 123.0
	WB 15%					
	SF 5%					
SMS 90%-HRL 10%	SMS 90%	26.9 ± 0.2	0.9 ± 0.0	27.2 ± 1.4	6.3 ± 0.7	730 ± 122.3
	HRL 10%					
SMS 80%-HRL 10%	SMS 80%	29.2 ± 1.2	1.3 ± 0.1	23.3 ± 0.9	6.5 ± 0.9	594 ± 50.9
	HRL 10%					
	WB 5%					
	SF 5%					
SMS 80%-HRL 20%	SMS 80%	26.6 ± 0.9	1.1 ± 0.2	25.3 ± 0.7	7.1 ± 0.5	624 ± 52.3
	HRL 20%					
SMS 70%-HRL 20%	SMS 70%	28.9 ± 0.2	1.5 ± 0.3	20.6 ± 1.1	6 ± 1.4	721 ± 64.3
	HRL 20%					
	WB 5%					
	SF 5%					
SMS 70%-HRL 30%	SMS 70%	26.3 ± 0.5	1.3 ± 0.1	21.2 ± 0.8	7.4 ± 0.5	756 ± 33.2
	HRL 30%					
SMS 60%-HRL 30%	SMS 60%	28.6 ± 0.4	1.7 ± 0.1	19.1 ± 1.4	6.6 ± 0.1	814 ± 73.2
	HRL 30%					
	WB 5%					
	SF 5%					
SMS 60%-HRL 40%	SMS 60%	26 ± 1.2	1.5 ± 0.1	19.9 ± 1.6	7.7 ± 0.0	1068 ± 27.6
	HRL 40%					
SMS 50%-HRL 40%	SMS 50%	28.3 ± 0.8	1.9 ± 0.1	17.2 ± 2.2	7.0 ± 0.5	1016 ± 65.8
	HRL 40%					
	WB 5%					
	SF 5%					
WS	WS 80%	33.3 ± 1.1	1.3 ± 0.2	25.1 ± 1.2	6.0 ± 0.2	561 ± 107.5
	WB 15%					
	SF 5%					

* Mean values with standard deviations from three replicates (±standard deviation).

**Table 2:** Sequences, reaction efficiency and amplicon sizes of oligonucleotide primers used in qPCR.

Gene (Accession Number)	Primer Direction	Sequence (5′-3′)	Reaction Efficiency	Amplicon Size
*B2M* (NM_004048.4)	Forward	GCTATCCAGCGTACTCCA	103	285
	Reverse	CTTAACTATCTTGGGCTGTGAC		
*RPS18* (NM_022551.3)	Forward	CTGAGGATGAGGTGGAACG	98	240
	Reverse	CAGTGGTCTTGGTGTGCT		
*RPL37a* (NM_000998.5)	Forward	AGTACACTTGCTCTTTCTGTGG	106	119
	Reverse	GGAAGTGGTATTGTACGTCCAG		
*NFE2L2* (NM_006164.5)	Forward	GATCTGCCAACTACTCCCA	90	121
	Reverse	GCCGAAGAAACCTCATTGTC		
*SOD1* (NM_000454.5)	Forward	CGAGCAGAAGGAAAGTAATGG	95	194
	Reverse	CCAAGTCTCCAACATGCC		
*CAT* (NM_001752.4)	Forward	TGCCTATCCTGACACTCACC	92	137
	Reverse	GAGCACCACCCTGATTGTC		
*HMOX1* (NM_002133.3)	Forward	GCTTCAAGCTGGTGATGG	90	112
	Reverse	AGCTCTTCTGGGAAGTAGAC		
*GSR* (NM_001195102.3)	Forward	CTTGCGTGAATGTTGGATGTG	98	102
	Reverse	CACAACTTGGAAAGCCATAATCAG		

**Table 3:** The effects of substrates consisting of spent mushroom substrate (SMS), the hydroponic roots of leafy vegetables (HRL) and control substrate (wheat straw—WS) on earliness, biological efficiency (B.E., %) and morphological parameters of *P. citrinopileatus.* Data are presented as mean values from duplicated measurements of six replicates (mean ± SD).

Substrate	Earliness (Days)	B.E. (%)	Pileus Diameter (cm)	Stipe Length (cm)
SMS 100%	30 ± 5	47.5 ± 2.2	5.2 ± 0.7	2.2 ± 0.0
SMS 90%	30 ± 5	45.9 ± 2.1	4.1 ± 0.2	1.9 ± 0.4
SMS 80%	30 ± 3	50.0 ± 1.9	3.7 ± 0.2	2.2 ± 0.3
SMS 90%-HRL 10%	30 ± 5	41.9 ± 3.6	4.6 ± 0.7	1.9 ± 0.4
SMS 80%-HRL 10%	26 ± 3	52.7 ± 2.4	4.1 ± 0.7	2.8 ± 0.0
SMS 80%-HRL 20%	26 ± 3	55.9 ± 2.8	4.1 ± 0.2	2.0 ± 0.4
SMS 70%-HRL 20%	27 ± 2	61.0 ± 4.1	4.4 ± 0.8	2.7 ± 0.2
SMS 70%-HRL 30%	26 ± 2	47.7 ± 1.8	5.2 ± 0.5	3.2 ± 0.2
SMS 60%-HRL 30%	30 ± 4	47.0 ± 1.5	4.3 ± 0.5	2.9 ± 0.8
SMS 60%-HRL 40%	27 ± 6	39.2 ± 3.2	3.7 ± 0.7	2.6 ± 0.2
SMS 50%-HRL 40%	27 ± 5	47.9 ± 3.4	5.1 ± 0.7	3.2 ± 0.3
WS	27 ± 6	73.5 ± 5.3	5.2 ± 0.7	2.0 ± 0.0

**Table 4:** Moisture, ash, total protein, lipid and carbohydrate contents (% *w*/*w* of dry biomass) of *P. citrinopileatus* cultivated on spent mushroom substrate (SMS), the hydroponic roots of leafy vegetables (HRL) and the control substrate (wheat straw—WS). Each point is the mean value of three independent measurements (mean ± SD).

Substrate	Moisture	Ash	Carbohydrates	Proteins	Lipids
	(%)	(%)	(%, *w*/*w*)	(%, *w*/*w*)	(%, *w*/*w*)
SMS 100%	89.9 ± 3.1	7.1 ± 0.9	62.6 ± 3.1	25.8 ± 1.2	4.5 ± 0.6
SMS 90%	90.3 ± 2.9	6.7 ± 0.8	60.9 ± 2.9	28.9 ± 1.1	3.5 ± 0.6
SMS 80%	89.4 ± 2.3	7.6 ± 1.0	61.4 ± 3.2	27.4 ± 0.9	3.6 ± 0.6
SMS 90%-HRL 10%	89.5 ± 2.4	7.6 ± 0.9	63.0 ± 2.5	25.6 ± 1.2	3.9 ± 0.3
SMS 80%-HRL 10%	89.0 ± 2.1	8.0 ± 1.1	57.0 ± 3.2	32.1 ± 1.1	3.0 ± 0.5
SMS 80%-HRL 20%	88.8 ± 2.3	8.2 ± 1.1	58.2 ± 3.1	31.0 ± 2.1	2.6 ± 0.5
SMS 70%-HRL 20%	89.7 ± 3.2	7.3 ± 0.9	62.9 ± 2.1	26.1 ± 1.4	3.6 ± 0.4
SMS 70%-HRL 30%	88.6 ± 1.9	8.4 ± 0.9	55.5 ± 2.4	32.5 ± 1.0	3.6 ± 0.2
SMS 60%-HRL 30%	88.6 ± 1.8	8.4 ± 0.7	56.2 ± 3.5	32.7 ± 0.9	2.8 ± 1.1
SMS 60%-HRL 40%	89.3 ± 2.9	7.8 ± 0.9	56.2 ± 3.2	31.9 ± 1.1	4.1 ± 0.9
SMS 50%-HRL 40%	88.8 ± 2.8	8.2 ± 0.8	52.0 ± 2.9	34.7 ± 0.8	5.1 ± 0.7
WS	90.4 ± 2.8	6.6 ± 1.0	65.7 ± 3.4	24.4 ± 1.0	3.3 ± 0.5

**Table 5:** Fatty acid composition (% *w*/*w*) of total lipids produced by *P. citrinopileatus* cultivated on spent mushroom substrate (SMS), the hydroponic roots of leafy vegetables (HRL) and control substrate (wheat straw—WS). Each point is the mean value of three independent measurements (mean ± SD).

Substrate/FA % *w*/*w*	C16:0	C16:1	C18:0	C18:1	C18:2	C20:0	C20:2	C22:0	C22:1	C22:6	C24:0	Other	Polyunsaturated	U.I.
SMS 100%	13.3 ± 1.0	0.5 ± 0.1	1.3 ± 0.2	6.2 ± 1.0	69.5 ± 2.1	0.6 ± 0.0	0.5 ± 0.1	0.5 ± 0.0	1.0 ± 0.1	0.8 ± 0.1	0.4 ± 0.1	4.3	78.5	1.5
SMS 90%	10.6 ± 0.9	0.3 ± 0.0	1.6 ± 0.1	6.0 ± 1.1	68.7 ± 2.0	0.6 ± 0.1	0.5 ± 0.1	0.4 ± 0.1	0.5 ± 0.0	0.8 ± 0.1	0.7 ± 0.1	7.3	76.7	1.5
SMS 80%	13.4 ± 1.3	0.2 ± 0.1	1.0 ± 0.1	6.4 ± 1.0	68.6 ± 3.0	0.4 ± 0.1	0.7 ± 0.0	0.6 ± 0.1	1.1 ± 0.1	0.8 ± 0.1	1.5 ± 0.1	4.7	77.6	1.5
SMS 90%-HRL 10%	10.9 ± 0.1	0.3 ± 0.1	1.1 ± 0.1	5.8 ± 1.1	68.4 ± 2.4	0.2 ± 0.1	0.6 ± 0.0	1.1 ± 0.1	0.6 ± 0.0	0.7 ± 0.1	0.3 ± 0.0	8.8	76.3	1.5
SMS 80%-HRL 10%	10.2 ± 0.1	0.5 ± 0.1	1.2 ± 0.1	4.6 ± 1.0	69.2 ± 2.4	0.4 ± 0.0	0.5 ± 0.1	1.0 ± 0.7	0.7 ± 0.1	0.9 ± 0.1	0.7 ± 0.0	8.9	76.3	1.5
SMS 80%-HRL 20%	10.1 ± 1.2	0.3 ± 0.0	1.2 ± 0.1	4.7 ± 0.9	63.8 ± 1.7	0.6 ± 0.0	0.7 ± 0.0	0.8 ± 0.0	0.9 ± 0.1	1.0 ± 0.1	0.6 ± 0.0	14.6	71.2	1.4
SMS 70%-HRL 20%	11.8 ± 1.1	0.3 ± 0.0	1.3 ± 0.2	5.5 ± 0.9	68.6 ± 1.9	0.5 ± 0.0	0.3 ± 0.1	0.7 ± 0.0	0.7 ± 0.1	0.6 ± 0.1	0.8 ± 0.0	7.7	76.0	1.5
SMS 70%-HRL 30%	13.6 ± 1.1	0.3 ± 0.0	1.0 ± 0.1	5.6 ± 1.2	74.4 ± 2.1	0.4 ± 0.1	0.6 ± 0.0	0.6 ± 0.1	0.8 ± 0.1	0.5 ± 0.0	0.6 ± 0.0	0.9	82.1	1.6
SMS 60%-HRL 30%	12.4 ± 1.0	0.3 ± 0.1	0.1 ± 0.1	4.8 ± 1.0	75.2 ± 2.4	0.4 ± 0.0	0.5 ± 0.1	0.6 ± 0.0	0.8 ± 0.0	0.6 ± 0.1	0.4 ± 0.0	2.3	82.1	1.6
SMS 60%-HRL 40%	11.2 ± 1.3	0.6 ± 0.1	1.2 ± 0.1	9.4 ± 1.0	66.3 ± 2.4	0.6 ± 0.1	0.5 ± 0.1	1.4 ± 0.0	1.3 ± 0.1	0.6 ± 0.0	0.9 ± 0.0	4.6	78.5	1.5
SMS 50%-HRL 40%	12.6 ± 0.5	0.29 ± 0.1	0.1 ± 0.1	5.0 ± 1.1	75.1 ± 1.9	0.2 ± 0.1	0.3 ± 0.1	0.2 ± 0.1	1.0 ± 0.1	0.7 ± 0.1	0.5 ± 0.1	1.9	82.5	1.6
WS	13.9 ± 0.1	0.3 ± 0.0	1.2 ± 0.1	6.9 ± 1.1	69.4 ± 2.5	0.3 ± 0.1	0.3 ± 0.0	0.7 ± 0.1	0.8 ± 0.1	0.6 ± 0.1	0.3 ± 0.0	3.8	78.4	1.5

**Table 6:** Carbohydrate composition (% *w*/*w*) of total IPSs produced by *P. citrinopileatus* cultivated on spent mushroom substrate (SMS), the hydroponic roots of leafy vegetables (HRL) and control substrate (wheat straw—WS). Each point is the mean value of three independent measurements (mean ± SD).

Substrate	Glucose	Fructose	Mannose
SMS 100%	43.9 ± 1.2	56.1 ± 1.2	nd
SMS 90%	40.5 ± 1.1	54.6 ± 1.7	4.8 ± 0.9
SMS 80%	37.9 ± 0.9	62.1 ± 2.3	nd
SMS 90%-HRL 10%	51.5 ± 2.1	48.5 ± 2.7	nd
SMS 80%-HRL 10%	29.5 ± 2.4	70.5 ± 3.1	nd
SMS 80%-HRL 20%	29.3 ± 1.0	70.7 ± 3.2	nd
SMS 70%-HRL 20%	18.5 ± 1.4	81.5 ± 1.2	nd
SMS 70%-HRL 30%	1.6 ± 0.5	98.4 ± 2.8	nd
SMS 60%-HRL 30%	7.7 ± 1.1	92.3 ± 2.2	nd
SMS 60%-HRL 40%	3.6 ± 0.9	96.4 ± 2.6	nd
SMS 50%-HRL 40%	12.8 ± 1.1	87.2 ± 1.9	nd
WS	30.0 ± 1.1	70.0 ± 1.7	nd

## Data Availability

All data presented in this paper are original for this study. The data used to support the findings of this study are available from the corresponding author upon request.
